# A preliminary mapping of QTL *qsg5.1* controlling seed germination in melon (*Cucumis melo* L.)

**DOI:** 10.3389/fpls.2022.925081

**Published:** 2022-08-15

**Authors:** Ling Wang, Junfeng Li, Fen Yang, Dongyang Dai, Xiang Li, Yunyan Sheng

**Affiliations:** College of Horticulture and Landscape Architecture, Heilongjiang Bayi Agricultural University, Daqing, China

**Keywords:** melon, QTL mapping, seed germination, phosphorus transporter PHO-5, gene analysis

## Abstract

Melon (*Cucumis melo* L.) seed germination significantly affects its economic value. Cultivation of melon varieties with high germination ability and seedling vigor is beneficial in large-scale melon propagation. In this study, two melon genotypes differing in their germination ability, P5 with low and P10 with high germination ability, were used to identify the optimal seed germination conditions by evaluating different water immersion times and germination temperatures. The germination rate of the P5 and P10 parental genotypes and their segregating population, consisting of 358 F_2:3_ families, were evaluated for 2 years to identify their genetic basis. QTL analysis was performed on a high-density genetic map constructed using specific-locus amplified fragment sequencing (SLAF-seq). The germination rate of F_1_ and F_2_ populations treated with water immersion for 8 h at 28°C and measured at 48 h showed a normal distribution Genetic mapping carried out using the high-density genetic map revealed eight QTLs in chromosomes 2, 4, 5, 6, and 8 that control melon seed germination, of which 2020/2021-*qsg5.1* was consistently significant in both years of experimentation. *qsg5.1* explained 15.13% of the phenotypic variance with a LOD of 4.1. To fine map the candidate region of *qsg5.1*, eight cleaved amplified polymorphism sequence (CAPS) markers were used to construct a genetic map with another 421 F_2_ individual fruits. The major QTL *qsg5.1* was located between SNP53 and SNP54 within a 55.96 Kb interval containing four genes. qRT-PCR gene expression analysis of the candidate genes showed that *MELO3C031219.2* (*Phosphorus transporter PHO-5*) exhibited a significant difference in gene expression between the parental lines at 24, 32, and 48 h after germination, potentially being the underlying gene controlling melon seed germination. These results provide a theoretical basis for the molecular mechanisms controlling melon seed germination and can practically contribute to further improving germination to increase the propagation efficiency of commercial melon cultivars.

## Introduction

Seeds are the most important means of agricultural plant propagation and a prerequisite for high-quality crop growth and development. Seed germination is the first stage of plant development, and high-quality and timely germination is important for crop yield and economic value (Nonogaki, 2017). Melon (*Cucumis melo* L., Cucurbitaceae) is an important cash crop that is widely grown worldwide and is primarily propagated with seeds. Melon has become a pillar crop for the agricultural industry and significantly contributes to the farmers' income in certain regions. Seed germination and dormancy under different environmental conditions have played a critical role in the survival of plants. Research on seed dormancy and germination recently showed that several factors are involved in seed germination (Nonogaki, 2014, 2017; Li W. et al., 2022; Nie et al., 2022). Extensive research has been conducted on the effects of different exogenous treatments on melon seed germination. Salt, alkali, germination initiation treatments, and hormone treatments significantly affect melon seed germination and seedling production (Barrero et al., 2009; Castañares and Bouzo, 2019; Saberali and Shirmohamadi-Aliakbarkhani, 2020; Zhang et al., 2022b).

In addition, temperature and water immersion time are two decisive factors for seed germination and presuppose the absorption of a minimum dose of water before the downstream metabolic activities are initiated (Debeaujon et al., 2007). Immersion time determines the degree of water uptake by seeds. Temperature is the most important external environmental factor affecting seed germination. An optimal temperature initiates the underlying physiological and biochemical response mechanisms and can maintain cellular homeostasis, thus yielding a maximum germination rate (Penfield, 2017). For most plants, seed germination can be promoted with increasing temperature; however, when the temperature rises to a certain level, seed germination becomes stagnant (Eberle et al., 2014; Zhang et al., 2015). Therefore, screening for the most suitable seed water immersion time and germination temperature is critical. Several transcription factors involved in seed dormancy have been identified in Arabidopsis (Verma et al., 2022). Transcriptional dynamics of two seed compartments during Arabidopsis seed germination indicated that large transcriptome changes mark the first germination phase of water uptake as the seed switches away from the desiccation state.

Seed germination is a complex process that is regulated by a combination of internal plant signals and external factors. Genes underlying seed germination have been identified by quantitative trait locus (QTL) analysis. In Arabidopsis, Delay of Germination 1 and Reduced Dormancy 5 genes have been identified by gene localization, regulating the level of seed dormancy via the gibberellin (GA) signaling pathway. The genetic control of five germination-related indexes, including germination percentage, was determined using quantitative trait loci (QTL) analysis in *Brassica napus*, providing insights into drought tolerance (Gad et al., 2021). He (2021) conducted a genetic and transcriptional analysis of seed germination in Chinese cabbage and identified genes related to flavonoid synthesis in the seed coat, embryo development, abscisic acid (ABA) synthesis, regulation of GA, and jasmonic acid (JA) synthesis during the seed germination process. Yang et al. (2021) fine mapped the *qLTG3-1* locus conferring low-temperature germination ability in rice to improve the efficiency and accuracy of trait selection during rice breeding. Han et al. (2014) identified six QTLs controlling seed germination traits under different temperature conditions in maize, with 35 candidate genes between their corresponding marker intervals mainly involved in seed germination, reduction-oxidation metabolic pathways, signaling, adversity, and other biological processes.

The SLAF-seq-based SNP marker identification is a powerful technique for the construction of high-density genetic maps (Geng et al., 2016; Li J. F. et al., 2022), and many high-density genetic maps have been constructed for melon (Wang et al., 2021), watermelon (Shang et al., 2016), and cucumber (Zhu et al., 2016). Using the SLAF high—throughput sequencing approach for genetic mapping and QTL detection, the genetic control of several traits has been elucidated, including fruit firmness (Dai et al., 2022), flowering-related traits (Wang et al., 2021), and stigma color (Du et al., 2022; Lv et al., 2022) in melon, as well as aphid resistance (Liang et al., 2016) in cucumber. The use of SLAF-seq in sweet corn to detect seed-related traits yielded 18 QTLs in two seasons (Wu et al., 2020).

So far, the studies conducted on QTL localization of seed-related traits in melons have mainly focused on 100-seed weight, seed length, and width (Lian et al., 2021; Zhang et al., 2022b). However, compared to other crops, only a few studies have been conducted on the genetic control of seed germination. Recently, Wu et al. (2022) identified the abscisic acid-insensitive (*ABI3*) transcription factor as one of the most important regulators of germination in cucumbers. The degradation of abscisic acid is critical for seed germination, as treatment with slightly acidic electrolyzed water inhibited ABA accumulation and improved the germination ability of watermelon seeds (Wu et al., 2020).

In this study, we selected melon genotypes that showed significant differences in seed germination traits and screened them to identify the optimal germination temperature and immersion time treatments. Using a high-density specific-locus amplified fragment sequencing (SLAF-seq) genetic map previously developed by our lab, we evaluated their segregating population to locate the seed germination traits and screen the main effect of QTLs. Our results provide insights into the molecular mechanisms underlying melon seed germination and can practically contribute to further improving germination to increase the propagation efficiency of commercial melon cultivars.

## Materials and methods

**Figure 1:**
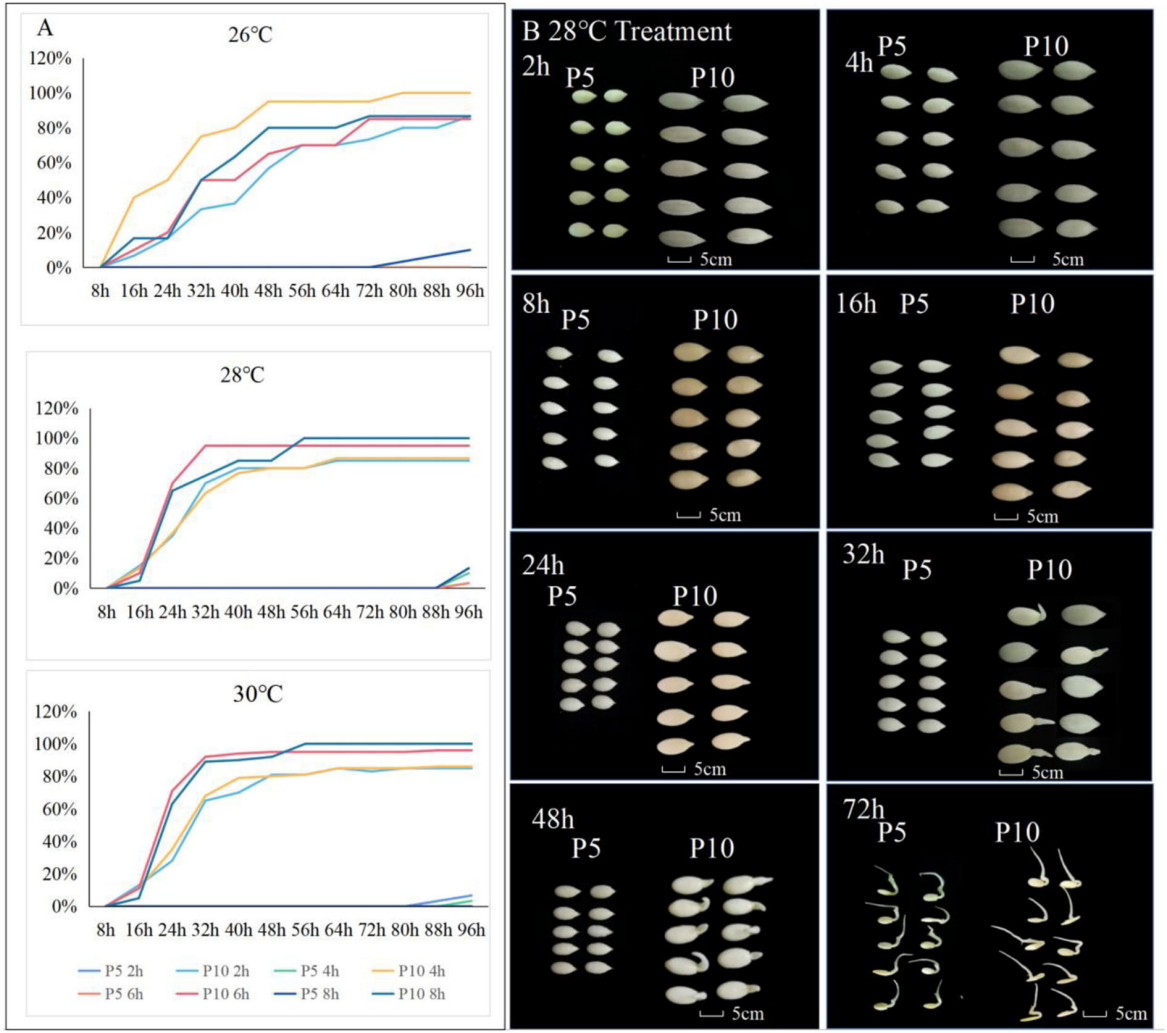
Seed germination performance for different treatments of germination temperature with soaking time in P5 and P10. **(A)** The dynamics of germination rate from 16 to 96 h after germination was investigated for the seed soaking time of 2, 4, 6, and 8 h with 26, 28, and 30°C germinate temperatures of parental lines. **(B)** The seed germination of P5 and P10 under the treatment with a soaking time of 8, germinate temperature of 28 h, and the investigated seed germination time from 2 to 72 h.

Two melon genotypes with significant differences in seed germination traits were evaluated. Genotype P5, the female parent, had a seed weight of 0.66 ± 0.05 g. The seed length and width were 4.73 ± 0.15 mm and 2.73 ± 0.10 mm, respectively. Pine-shaped seeds with yellow seed coats were primarily dormant. Their germination rate was 0 at 28°C after 8 h of immersion in water. Genotype P10, the male parent, had a seed weight of 1.35 ± 0.42 g, while seed length and width were 7.70 ± 0.93 mm and 3.74 ± 0.19 mm, respectively. Oval-shaped seeds with a white seed coat germinated easily. The germination rate exceeded 98% at 28°C after 8 h of immersion in water (Figure 1). The two parents were crossed to obtain F_1_, while self-pollination was used to obtain F_2_ individuals and F_3_ families.

To identify candidate gene expression patterns of *MELO3C031219.2*, different tissues of five melon subspecies (*ssp.melo*) during germination were also examined, including ZT091 (*ssp.agrestis*), M2-10 (*ssp.agrestis*), 1244 (wild, *ssp.agrestis*), ms5 (*ssp.melo*), and Bobo (*ssp.melo*).

### Investigation of seed germination rate

The parental seeds were selected from the current year's harvest and then immersed for 2, 4, 6, and 8 h in distilled water (50°C). The germination was carried out at different incubation temperatures (26, 28, and 30°C) in a cabinet with constant temperature and humidity (70%).

In total, 12 treatments were used to investigate the germination rate of the parents at 8-h intervals from 16 to 96 h after germination. The germination dynamics of the parents were subsequently analyzed. In each treatment (T), 100 randomly selected seeds from the parental lines were evaluated in three replicates, using a completely randomized design (CRD). The germination rate for each treatment was recorded and calculated as a percentage. The optimal immersion time, germination temperature, and the time points during which significant differences in germination between the parents were observed are depicted in Table 1.

**Table 1:** Treatment of soaking time and immersion temperature.

**Treatment**	**Temperature**	**Soaking time**
T_1_	26°C	2 h
T_2_		4 h
T_3_		6 h
T_4_		8 h
T_5_	28°C	2 h
T_6_		4 h
T_7_		6 h
T_8_		8 h
T_9_	30°C	2 h
T_10_		4 h
T_11_		6 h
T_12_		8 h

### SLAF-seq-based genetic map construction

Leaves from the parents and 120 F_2_ individuals were sent to Beijing Biomark Biotechnology Co., Ltd. for SLAF-seq. The melon genomic DNA of parents and the F_2_ generation was extracted using the modified cetyltrimethylammonium bromide (CTAB) method (Nguyen et al., 2018).

Based on the resequencing of parental lines, single-nucleotide polymorphisms (SNPs) were identified between parents. SNP detection in the F_2_ plants was mainly implemented using the GATK software package (https://wiki.rc.usf.edu/index.php/Genome_Analysis_ToolKit_GATK). Genetic maps were constructed using the modified logarithm of odds (MLOD) between the SNP markers. The markers were arranged linearly, and a genetic map with 12 linkage groups was generated using the High Map software (http://highmap.biomarker.com.cn/).

### QTL localization analysis of seed germination traits

The QTL analysis of seed germination traits was performed using the high-density linkage map constructed in our lab, using the SLAF sequencing technology. The genetic map was constructed after sequencing 662,772 SLAF markers. It contained 12 linkage groups generated from 3,716 genetic markers, with a total length of 1,356.49 centiMorgan (cM). The average genetic distance between the markers was 0.37 cM (Li J. F. et al., 2022).

The seeds of F_3_ populations were harvested and dried in 2020 and 2021, respectively. The F_2_ population seeds were harvested in 2021, and their germination rates were evaluated by immersing the seeds in water for 8 h at 28°C. The germination rates were calculated at 48 h after the seeds started to germinate. For each of the parental lines, P_1_ and P_2_, 100 seeds from each fruit were measured, and the average value of three replications was calculated. For SLAF-QTL mapping, seed germination rates of F_3_ families were determined, and the mean values were also calculated as the performance of F_2_ in 2020 and 2021, respectively. For the F_3_ population, 358 families were investigated, with ~10 fruits collected from 10 individuals of each family and ~100 seeds collected from each fruit.

Based on phenotypic data, QTL analysis was carried out using the IciMapping method to map the preliminary QTL locations of seed germination. Threshold values were set by CIM interval mapping and 1,000 times permutation tests (PTs). The LOD threshold was set at 2.5. The terminology used for naming the QTL was QTL+Trait English abbreviation +linkage group number + QTL number.

### CAPS marker development to fine map the candidate QTL region of *qsg5.1*

To narrow the candidate region of *qsg5.1*, another 421 F_2_ individuals were investigated, and ~200 seeds from each fruit were evaluated for QTL localization analysis. Cleavage amplified polymorphic sequence (CAPS) markers were developed based on the resequencing data from parental lines P5 and P10 (Supplementary Table 1). Primers for CAPS markers were designed with Primer Premier 5.0 (http://www.premierbiosoft.com/) and CAPS Finder 2.0 (http://helix.wustl.edu/dcaps/dcaps.html). Polymerase chain reaction (PCR) was carried out with 20 ng of template DNA, 1.0 μM of each forward and reverse primers, 0.2 mM of dNTP mix, 0.1 U of Taq DNA polymerase, and 1 × PCR buffer (Takara, China) in a total volume of 10 μL. PCR products were digested with restriction enzymes after incubating for 4 h at the optimal temperatures designated by the manufacturer. The resulting DNA fragments were separated using a 6% polyacrylamide gel with silver staining (Sheng et al., 2017).

### Candidate gene identification

Melon DNA sequences were retrieved from the http://cucurbitgenomics.org/organism/18 database (DHL92) v3.6.1 Genome. The sequencing products were queried to the database using blast (http://blast.ncbi.nlm.nih.gov/Blast.cgi/) to identify the gene annotation.

### Candidate gene expression pattern analysis at the primary locus

The parental seeds were immersed in water for 8 h. Afterward, the seeds were placed in an incubator box, and they (including sprouted shoots) were sampled at 24, 32, and 48 h for qRT-PCR to measure the levels of gene expression. RNA was extracted using the Polysaccharide Multifraction Plant Total RNA Extraction Kit (Tiangen, China). The cDNA was obtained by reverse transcription using a PrimeScript™ RT reagent kit with a gDNA Eraser kit (Takara, RR047A) and stored at−20°C. cDNA sequences of each annotated gene in the candidate locus interval were used to develop primers using primer 3 (http://www.primer3plus.com/cgi-bin/dev/primer3plus.cgi) and synthesized by the Shanghai Biotech company. Real-time PCR (qRT-PCR) was performed according to the instructions of Takara's SYBR Green PCR kit (Takara, RR420A).

The reaction was carried out in a reaction mixture containing 10 μL of template cDNA (100 ng/μL), 5 μL of 2 × SYBR Green PCR Master Mix, 0.75 μL of forward and reverse primers (10 μM), and 2.5 μL of ddH_2_O. The PCR amplification procedure was as follows: 95°C for 30 s pre-denaturation; 95°C for 15 s, 58°C for 30 s, 72°C for 60 s, 40 cycles. Three biological replicates were used for all samples, with three technical replicates for each biological replicate. The analysis included biological and technical replicates, with reference genes used as internal reference (Wang et al., 2021). Differential expression was determined using the 2^−ΔΔCt^ method.

### Data analysis

The field data were analyzed using the SAS program (SAS Institute Inc). A normal distribution test was performed on the seed-related traits of the parents and the F_2_ population using the software Origin 2019. The phenotypic data were used for QTL analysis. QTLs for each trait were identified using the composite interval mapping (CIM) method. The threshold was set by conducting a PT test with 1,000 iterations and a LOD value of 2.5. The terminology used for naming the QTL was “trait + chromosome number + QTL number.”

To compare the differences in the genetic sequence of the candidate gene *MELO3C031219.2*, the resequencing data from the parental lines and five additional melon lines were used for analysis by DNAMAN Version 9.

## Results

### Parental seed germination

The results obtained for the different treatments indicated that the parent P5 did not germinate in (Treatment) T_1_, T_2_, and T_3_.treatments. In T_4_, the seeds started to germinate after 80 h with a germination rate of 3% and reached 10% in 96 h. In treatments T_4_-T_8_, the germination performance of parent P5 at 96 h increased with the immersion time; thus, seed germination increased by 3% (T_5_), 10% (T_6_), 11.6% (T_7_), and 13% (T_8_), respectively. In the treatments T_9_-T_12_, using a water immersion temperature of 30, the germination rate decreased as the immersion time increased. In the 2-h immersion treatment (T_9_), seeds germinated 88 h after the seed germination treatment, and only 3% of seeds were broken, while in 96, only 7% of seeds germinated. In the 4-h water immersion treatment (T_10_), seeds began to germinate after 96 h. On the contrary, in the 6-h (T_11_) and 8-h (T_12_) immersion treatments, seeds of parent P5 did not germinate even after 96 h.

The male parental line P10 performed well in terms of seed germination. In the 2-h water immersion treatment with an immersion temperature of 26°C (T_1_), the germination rate was 7% at 16 h and reached 87% at 96 h. In the T_2_ treatment, 10% of the seeds germinated at 6 h and reached 85% at 72 h. About 10% of the seeds germinated in the T_3_ treatment after 16 h and reached 85% after 96 h. After 8 h of immersion at a temperature of 26°C, the germination rate was 17% at 16 h and 87% at 56 h. In the T_5_-T_8_ treatments, seeds of P10 germinated in 16, but the germination rate decreased as the immersion time increased (15% in 2, 13% in 4, 10% in 6, and 5% in 8 h). This indicated that at an immersion temperature of 28, extending the immersion time did not increase the seed germination in P10. In the T_5_ treatment, the germination rate was 15% at 16 h and reached 65% at 24, with a continuously increasing trend, and reached 100% at 56 h. In the T_6_ treatment, germination started at 16 h with a rate of 13% and reached 90% at 64, while in T_7_, 10% of seeds germinated at 28°C at 16 h and reached 95% at 32 h. In the T_8_, only 5% of the seeds germinated in 16 h but rapidly reached 100% germination after 56 h. At a water immersion temperature of 30°C, germination was initiated at 16 h with a rate of 10%, reaching 95% at 64 h. The percentage of seeds germinating at 30°C was 10% at 16 h and 95% after 32 h. At 30°C, the same trend observed at 28°C was noticed, irrespective of immersion time; the seeds began to germinate in 16, but with the extension of immersion time, the germination rate decreased. In the T_9_ and T_10_ treatments, the germination rate of seeds reached ~85% at 56, and this germination rate remained the same till the 96-h time point. In the T_11_ and T_12_ treatments, the seed germination rate reached more than 90% at the 32-h time point (Figure 1).

### Differences in germination rates of parents

To clarify the significance of differences in the germination rates of parental seeds, the germination rates of parental seeds under different immersion times and treatment temperatures were assessed. No significant differences were observed in the germination rates between the parents in the 2-h and 4-h treatments (Table 2). The germination rates between parents differed significantly after 6 h of immersion at 30°C and 48 h of treatment. In addition, 8 h of immersion at 28°C resulted in significant differences in germination rates between parents at 48 h and 56 h (Table 2). Therefore, according to our results, the optimal seed germination was achieved with the water immersion time of 8 h and at a temperature of 28°C (treatment T_8_).

**Table 2:** Significant difference analysis of seed germination rate between parental lines.

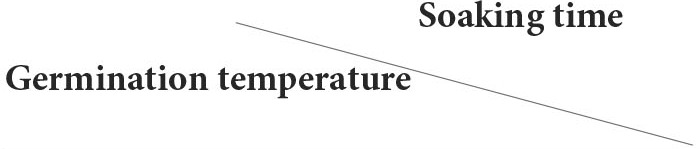	**26** **°** **C**					**28** **°** **C**					**30** **°** **C**				
	**Investigated time**					**Investigated time**					**Investigated time**				
	16 h	24 h	32 h	48 h	56 h	16 h	24 h	32 h	48 h	56 h	16 h	24 h	32 h	48 h	56 h
2 h	0.082	0.292	0.221	0.369	2.100	0.112	0.186	0.318	0.362	0.189	0.236	0.229	0.471	0.471	0.429
4 h	0.327	0.346	0.364	0.449	2.000	0.149	0.297	0.362	0.428	0.102	0.329	0.323	0.428	0.428	0.105
6 h	0.082	0.121	0.292	0.325	1.600	0.075	0.335	0.449	0.428	0.052	0.236	0.229	0.468	0.044^*^	0.028^*^
8 h	0.098	0.098	0.306	0.446	2.650	0.400	0.381	0.338	0.037^*^	0.047^*^	0.330	0.371	0.484	0.032^*^	0.012^*^

* Means significant level P < 0.05.

### QTL localization of seed germination

The seeds of female parent P5 remained dormant after 8 h of immersion time and treatment temperature of 28°C, with the germination rate remaining 0% within 48 h of treatment initiation. In contrast, the seeds of the male parent P10 started to germinate at 24, with an average germination rate of 98% at 48 h and a range of 97–100%. The F_1_ seeds started to germinate at 24, with an average germination rate of 85% at 48 h and a range of 55–91% (Table 3). Thus, in the F_1_ hybrid, the germination rate of P5 × P10 was better than that of the individual parents. The germination started after 8 h under different germination temperatures and seed immersion treatments, and reached 100% within 24–32 h.

**Table 3:** The germination rate in 48 h after germination for melon seeds under the treatment of 28°C with 8 h soaking.

**Group**	**Germination rate**			
	**Average**	**SD**.	**Variation**	**CV**
Fmale parent-P5	0	0	0	0
Male parent-P10	98%	0.45	97–100%	1.21
F_1_	85%	2.52	83–87%	13.68
F_2_	63%	7.48	0–100%	25.24

**Figure 2:**
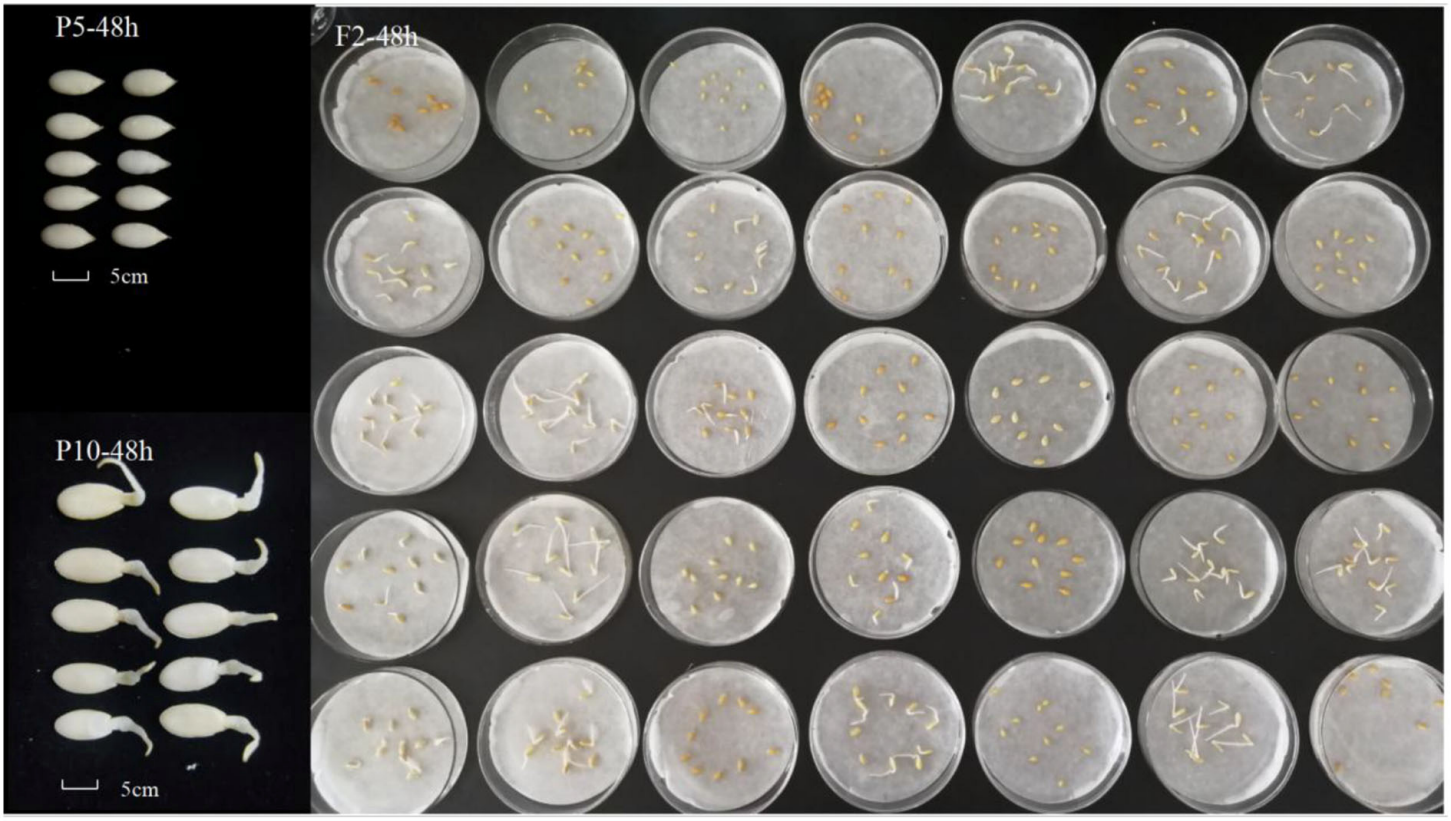
Seed germination performance of two parents and their F_2_ populations under the treatment with soaking time of 8 h and germinate temperature of 28°C. Seeds from F_2_ individuals fruit germination rate were investigated in 48 h, and each culture dish contains one fruit seed.

The F_2:3_ populations were obtained by selfing. A genetic analysis was conducted on the newly harvested seeds to investigate the average germination performance of each of the 358 F_3_ family seeds. The distribution of the 2-year germination rate is shown in Figure 2. The germination rate of the population at 48 h exhibited a normal distribution, with 19 and 13 lines having a germination rate of <10% in 2020 and 2021, respectively, while 35 and 30 lines had a 100% germination rate in both years. Therefore, the distribution indicated that the seed germination rate is under quantitative genetic control with major loci controlling a significant percentage of the variation (Figure 2).

**Figure 3:**
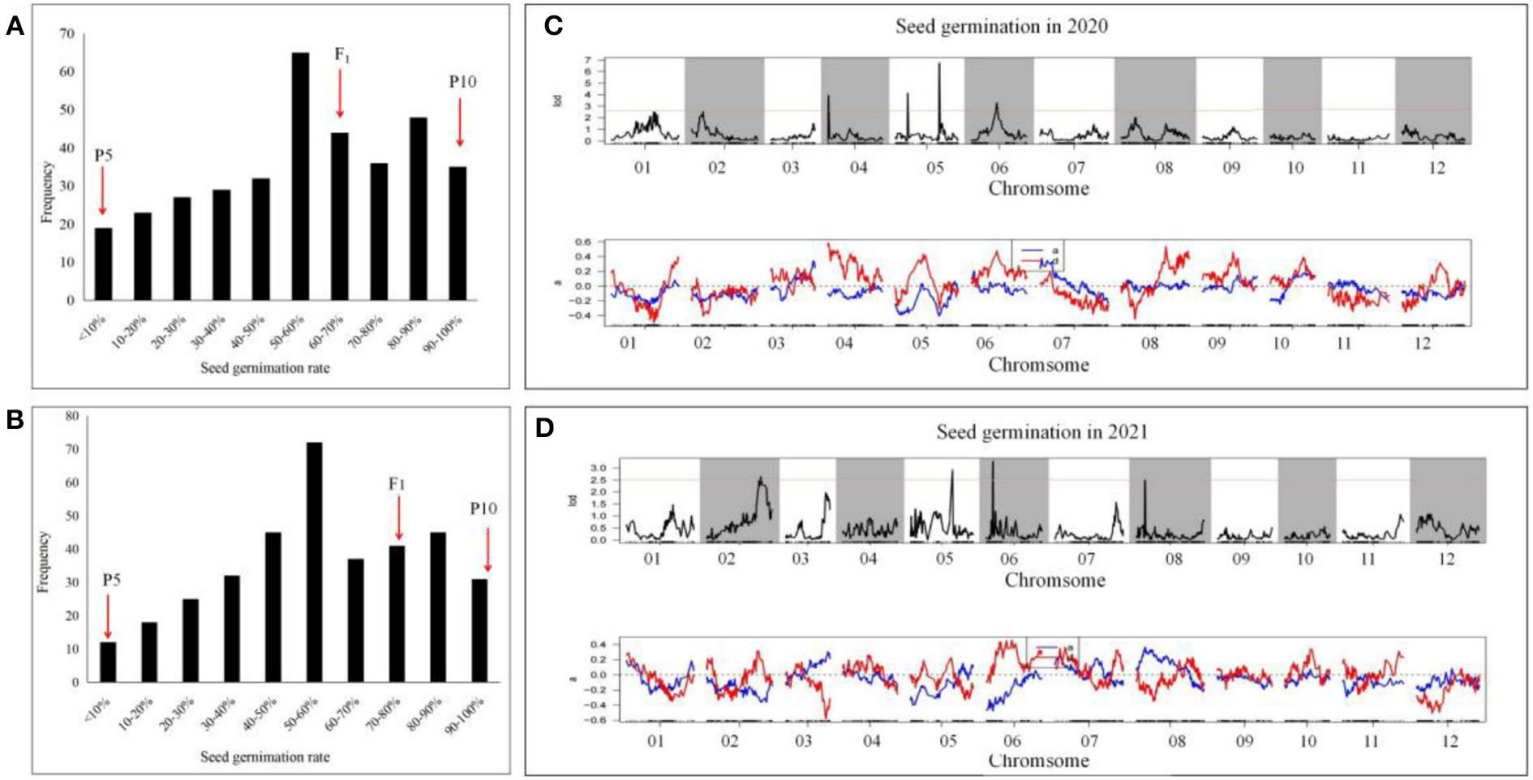
Seed germination segregation of F_2:3_ generation and QTL preliminary detection using previously constructed genetic map. The average data for seed germination of F_2:3_ families with each family containing 10 fruits were collected in 2020 and 2021, respectively. **(A,B)** the distribution of seed germination in 2020 and 2021. **(C,D)** QTL detection for seed germination in 2021 and 2021. For the upper map, numbers to the abscissa axis was each chromosome (Chr) and the map length in centiMorgan (cM), and the vertical bars represent 2.5 LOD support interval of each QTL. For the blow map, those indicated the additive effects for the QTL loci.

Using the previously developed SLAF genetic map, QTL analysis was carried out to map the loci controlling the seed germination traits of *Cucumis melo* L. The results showed that seed germination in 2020 was a quantitative trait, with four QTLs located on chromosomes 4, 5, 6, and 8 of the QSG (Table 4; Figure 3). Among them, 2020-*qsg4.1* was located on chromosome 4, between 2,073,104 and 2,609,033 bp, within the linked markers 282,269 and 284,109. Its LOD value was 2.5; the QTL explained 7.1% of the variance, and the additive effect was −0.41. Two QTLs were located in chromosome 5, one in the short arm and the other near the end of the chromosome. Specifically, 2020-*qsg5.1* was located at a genome position between 5,372,818 and 5,406,648, linked with markers 672,238 and 672,241. It had a LOD value of 6.7, explained 10.52% of the total variance, and had an additive effect of −0.408. *2020-qsg5.2* was located between 4,610,044 and 4,657,221 bp in chromosome 5, a much larger region of 4,718 Kb. Markers 667,064 and 667,434 were linked with the QTL, the LOD threshold was 4.2, and the QTL explained 10.52% of the total phenotype variance. The additive effect was 0.08, indicating that the effects of this locus to promote early seed germination were contributed by the male parent. Furthermore, 2020-*qsg6.1* was located on chromosome 6, between 727,434 and 874,956 bp, with markers 808,517 and 820,096 at each end. It had a LOD value of 32, explained 5.8% of the variance, and had an additive effect of−0.47. Its QTL interval had a total length of 147.52 Kb.

**Table 4:** QTL analysis of seed germination rate using F_2_ population obtained by crossing P5 with P10.

**QTL ID**	**Chr.^a^**	**Genetic map position (cM)**		**Position of genome**	**Region marker**		**LOD**	**PV^b^**	**Add^c^**
*2020-qsg4.1*	4	21.53	26.57	2073104-2609033	Marker282269	Marker284109	2.5	7.10%	−0.41
*2020-qsg5.1*	5	83.50	83.92	5372818-5406648	Marker672238	Marker672741	6.7	14.15%	−0.4
*2020-qsg5.2*	5	26.83	36.89	4610044-4657221	Marker667064	Marker667434	4.2	10.52%	0.08
*2020-qsg6.1*	6	48.72	56.06	4541536-7591934	Marker808517	Marker820096	3.2	5.80%	0.47
*2021-qsg2.1*	2	103.43	104.27	24860953-25058913	Marker381496	Marker382095	2.7	7.74%	−0.34
*2021-qsg5.1*	5	83.51	83.92	5305884-5406648	Marker670274	Marker672741	3.0	13.08%	−0.37
*2021-qsg6.1*	6	12.203	13.044	727434-874956	Marker794758	Marker793699	2.6	10.02%	−0.47
*2021-qsg8.1*	8	16.808	17.228	1742525-1779289	Marker1020658	Marker1018039	2.51	9.21%	0.36

a Indicates chromosome;

b Indicates prototypical variance; and

c Indicates additive effects.

When QTL analysis was performed on the data collected in 2021, four QTLs were identified, which were located on chromosomes 2, 4, 5, and 6. *2021-qsg2.1* was located on chromosome 2; its position was between 24,860,953−25,058,913 bp, with 177.96 Kb interval. It was linked with markers 381,496, 382,095, had a LOD value of 2.7, explained 7.7% of the phenotypic variance, and its additive effect was −0.34. The QTL *2021-qsg5.1* was located on chromosome 5, between 5,305,884 and 5,406,648 bp, a 100.77 Kb interval, with markers 670,274 and 672,241 closely linked. Although its LOD value was only 3.0, it explained 13.08% of the phenotypic variance. The additive effect of *2021-qsg5.1* was −0.372, indicating that the male parent contributed to its phenotypic effect. QTL *2021-qsg5.1* was close to *2020-qsg5.1*, which was identified in 2020; however, its interval was larger than *2020-qsg5.1*, but both shared the same end position marked by marker 672,241. QTL *2021-qsg6.1* was located on chromosome 6, between 727,434 and 874,956 bp, a 147.5 Kb interval. The LOD threshold of this QTL was 2.6 and explained 10.02% of the phenotypic variance. QTL *2021-qsg8.1* was located on chromosome 8, between 1,742,525 and 1,779,289 bp, delineated by markers 1,020,658 and 1,018,039 at each end. It had a LOD value of 2.5, explained 9.2% of the phenotypic variance, and it had an additive effect of 0.364 (Table 4; Figure 3).

### Mapping the candidate region of *qsg5.1*

**Figure 4:**
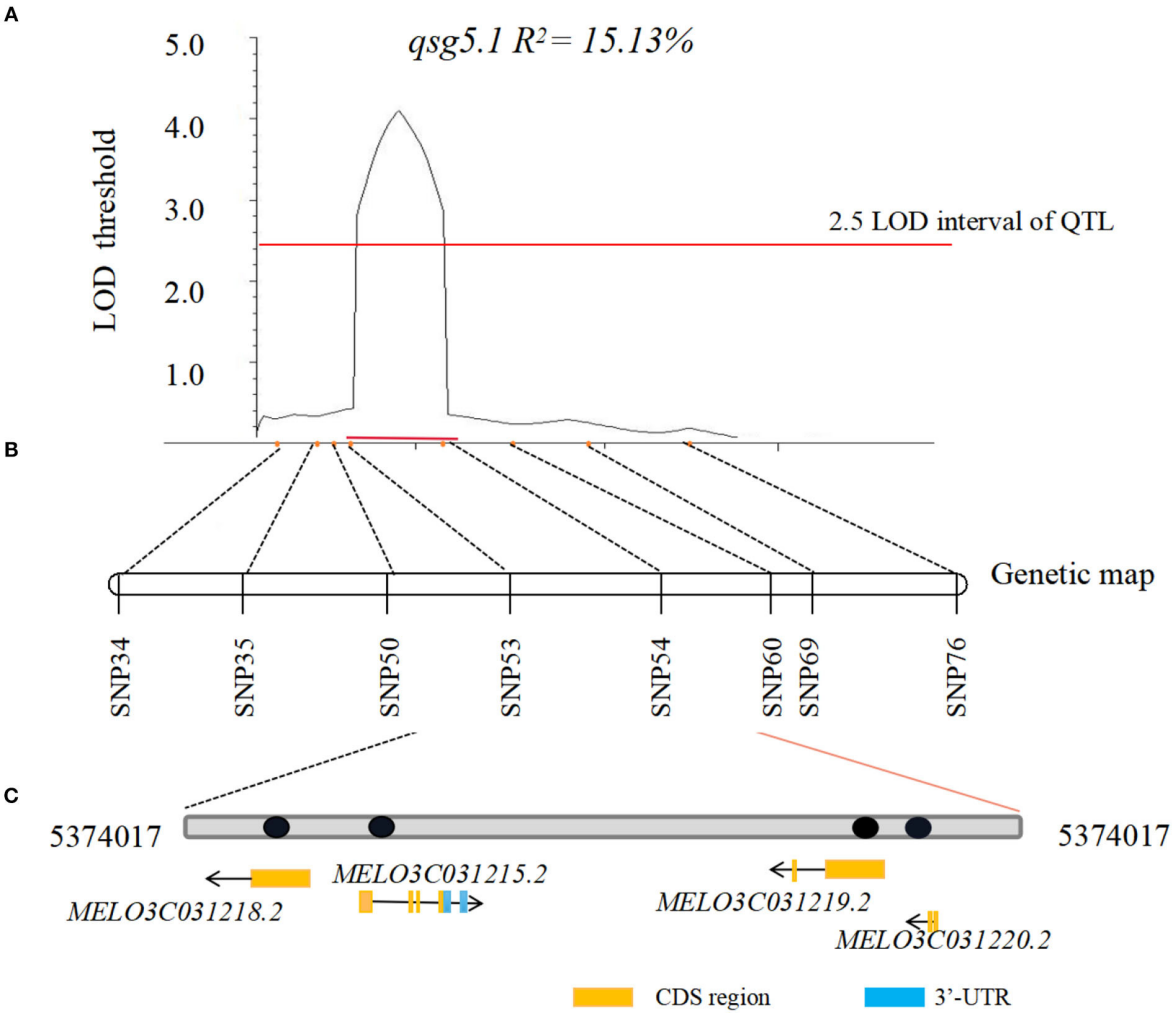
LOD curves of QTL for seed germination detected in a large F_2_ population. **(A)** Fine mapping based on consistent QTL *qsg5.1* for seed germination rate was detected with 421 F_2_ plants, and phenotype of each F_2_ individual fruit of 2021 defined the candidate locus to a 33.83 Kb region between markers SNP53 and SNP54. Horizontal dashed lines are LOD threshold values for declaring significant QTL. Horizontal red bars define 2.5 LOD interval of QTL. The percentage of phenotypic variations explained by each QTL (R^2^) is shown. **(B)** Genetic map construction is based on preliminary SLAF-seq QTL region. **(C)** Candidate genes in interval region which contain four genes.

When the QTL loci between the 2 years were compared, the major *2020/2021-qsg5.1* was the only QTL detected in 2020 and 2021. Thus, its effect on seed germination rate was stable and not affected by the year and the environment. To narrow the candidate region, additional fruits from 421 F_2_ individuals were assessed (each fruit from one plant investigated contained ~300 seeds). Eight CAPS markers were used to construct a more detailed genetic map between genome positions 3,419,909 and 7,602,535 bp on chromosome 5. This major QTL was located between SNP53 (5,374,017) and SNP54 (5,424,974), with the 50.96 Kb interval region containing four annotated genes (Table 5, Figure 4). Its LOD threshold was 4.1, and this QTL explained 15.13% of the phenotypic variance. We used the *Cucumis melo* L. genome database information (http://cucurbitgenomics.org/) to narrow down the candidate genes for the *Cucumis melo* L. seed germination trait. The interval of the locus *qsg5.1* contained four genes, including two genes of unknown function, a transmembrane protein, and a PHO-5 protein family, a phosphorus transporter gene (Table 1; Figure 3).

**Table 5:** Candidate gene sequencing and gene annotation for QTL *qsg5.1*.

**NO**.	**Gene ID**	**Gene annotation**	**Forward primer sequence**	**Reverse primer sequence**
1	MELO3C031215.2	Unknown protein	TGCGAGGAAAAGGAAACGGAA	ATAGTCGGCCAGCAAAAGTCA
2	MELO3C031218.2	Transmembrane protein	TGCCATTCTCTGCCACCTAC	ACCGCATGAACTCCCATTGA
3	MELO3C031219.2	Phosphate transporter PHO1-like protein 5	CGGGAGGCTTCTCATGGATT	ACTGCACCTGACTGAACGAA
4	MELO3C031220.2	Unknown protein	CCCAACTGTTTGATTACGCCC	TCACAATCCAAGGTTCCGCA
5	actin		GGTGATGAAGCTCAGTCCAA	TGTAGAAGGTGTGATGCCAAA

**Figure 5:**
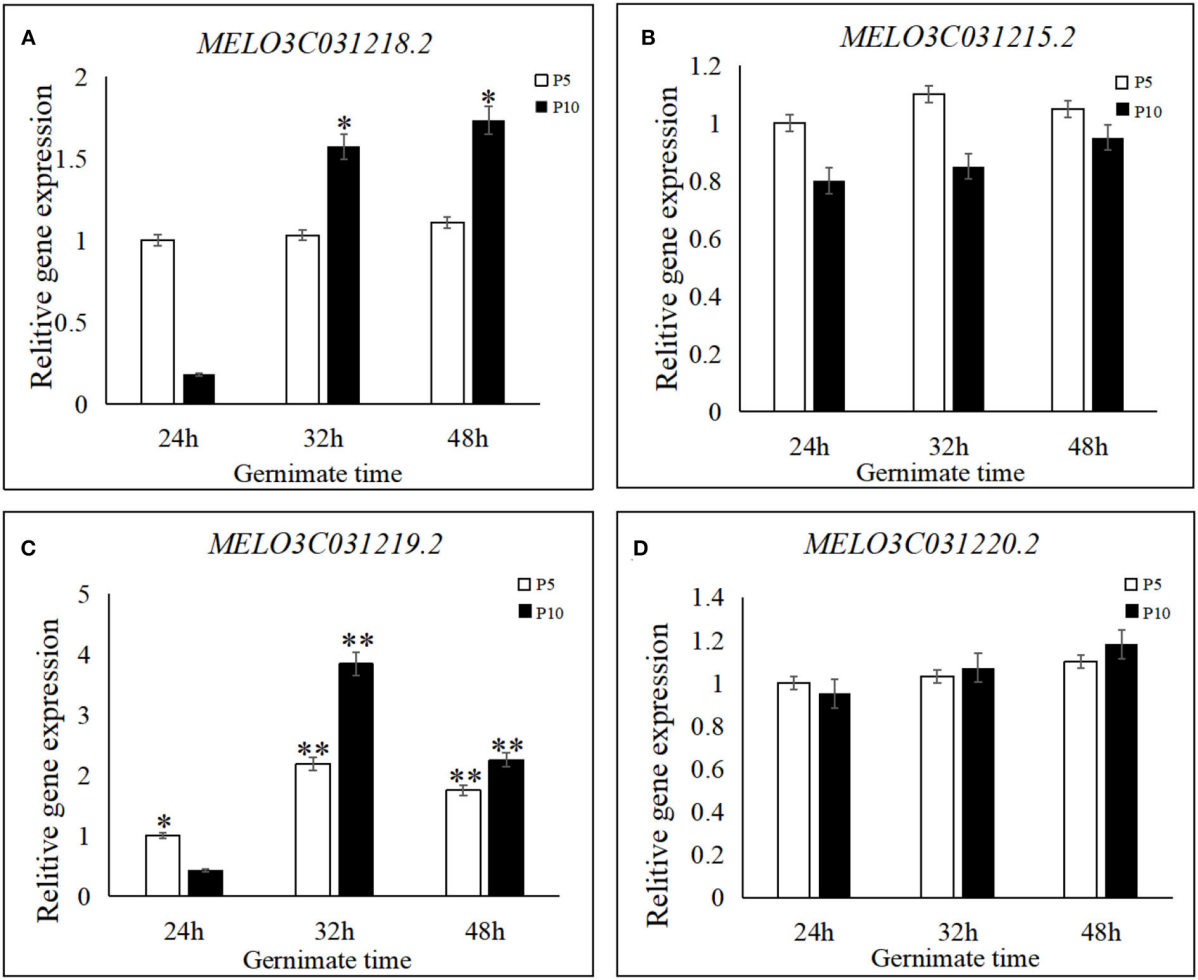
Relative expression levels of four candidate genes in seed germination for “P5” and “P10.” **(A)** Expression levels of *MELO3C031218.2*, **(B)** expression levels of *MELO3C031215.2*, **(C)** expression levels of *MELO3C031219.2*, and **(D)** expression levels of *MELO3C031220.2*. Candidate genes were quantified using the 2^−ΔΔCT^ method. For the two parents, the expression level of the respective genes at 24, 32, and 48 h after germination following treatment by 8-h soaking at 28°C. Each experiment was repeated three times, and 15 plants were mixed in equal amounts to form one replicate in two parents. **indicates an extremely significant difference, *P* < 0.01; *indicates a significant difference, *P* < 0.05.

To examine the expression levels of their genes, three stages of seed germination were assessed in the parental lines (Figure 5). No significant differences were observed between the two unknown genes, *MELO3C031215.2 and MELO3C031220.2*, between parental lines and the three seed germination stages, while their gene expression was moderate to low. Although a slight increase was detected in *MELO3C031220.2* between P5 and P10 after 24, 32, and 48 h of treatment, it was not statistically significant. The gene expression level of *MELO3C031215.2* was almost similar in both P5 and P10 and the three germination stages. The expression of the *MELO3C031218.2* gene in the parental line did not exhibit significant differences among the three seed germination stages. On the other hand, a significant difference was detected in parental line P10, which showed an increase in gene expression with an increase in seed germination time. Based on these results, *MELO3C031218.2* may be responsible for controlling the observed differences in the seed germination process; however, further research is needed to prove this hypothesis.

On the other hand, *MELO3C031219.2* expression was significantly different among the three germination stages and between the parental lines P5 and P10. Its expression was initially increased and then decreased at 24, 32, and 48 h in P5, and the same trend was also detected in P10. When comparing the parental lines, significant differences were also detected in each germination stage. As shown in Figure 5, the 32-h time point was key for seed germination, as seed germination sharply increased and most seeds germinated in 32 h. Therefore, our results indicate that the *MELO3C031219.2* gene, which encodes for a functional phosphate transporter PHO1-like protein 5, is a candidate gene for the control of seed germination in melon.

### DNA structure analysis and gene expression patterns in other melon genotypes

**Figure 6:**
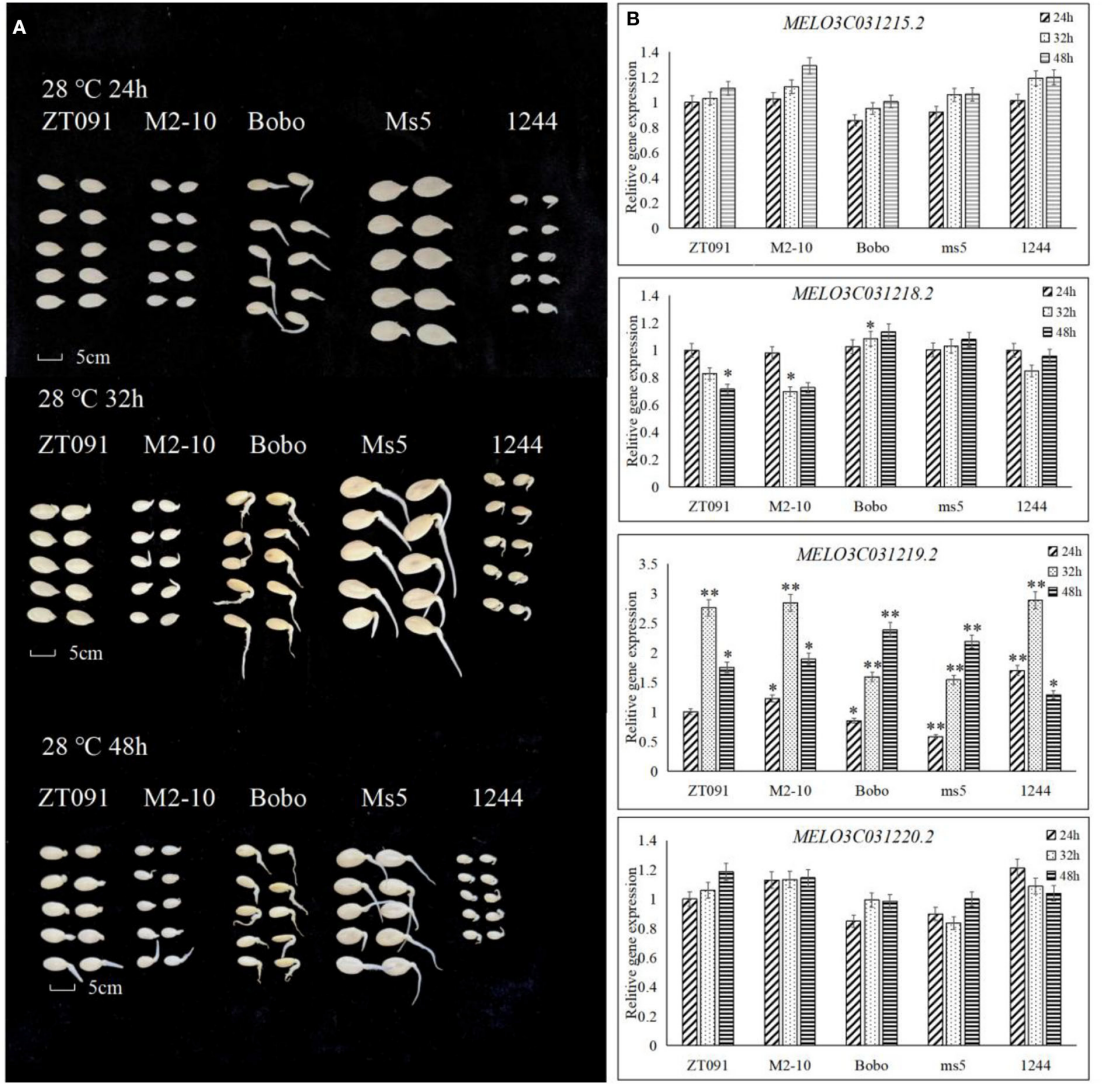
Performance of the germination of other five melon seeds and candidate gene expression. **(A)** The seed germination of five melon germplasms under the treatment including a soaking time of 8 h with the germinate temperature of 28°C and the investigated seed germinate rate at 24, 32, and 48 h. **(B)** Gene expression pattern in candidate interval by qRT in different seed germinate stages. * means significant level *P* < 0.05. ** means significant level *P* < 0.01.

To confirm the expression patterns of candidate genes, five melon genotypes were used to conduct qRT-PCR at the different germination stages (Figure 6). Regarding their phenotypic response, ZT091 (*ssp.agrestis*) and M2-10 (*ssp.agrestis*) did not germinate in 24 h but their germination was initiated at 32 h. However, for the other three melon genotypes Bobo (*ssp. melo*), ms5 (*ssp. melo*), and 1,244 (wild, *ssp. melo*), germination started within 24 h. At 48, seeds from all these melon genotypes germinated more than 50%. Based on the expression levels of candidate genes, *MELO3C031215.2* and *MELO3C031220.2* did not show significantly different expression levels in the different seed germination stages among the additional five melon lines evaluated. For *MELO3C031218.2*, different expression levels were detected among ZT091, ms5, and Bobo at 32 h during seed germination. However, there were no significant differences in ZT091 and ms5, and no consistent trend with regard to expression was observed in the five melon genotypes. In four melon genotypes (ZT091, M2-10, Bobo, and ms5), *MELO3C031218.2*, gene expression pattern showed an increase from 24 to 48, but this was not observed in genotype 1,244. In genotypes ZT091, M2-10, and 1,244, the gene expression level initially increased and then decreased. Significant differences in the gene expression were detected at the 24 h seed germination stage, while at 32, no differences were observed among the genotypes, except for Bobo and ms5. At the 48-h time point, gene expression was the highest in Bobo and lowest in ms5, and the difference was found to be significant.

Based on the parental resequencing data analysis, *MELO3C031219.2* is located on Chr05, with coordinates at 5,411,284–5,413,754 bp. Its CDS region is 1,686 bp in length, and three non-synonymous mutations were identified between the parents, which were located at 5,412,715 bp with an A to C mutation, 5,412,885 bp (A to C), and 5,413,009 bp (G to A) (Supplementary Figure 1). All these mutations were present only in the sequence of the parental line P5. These mutations result in changes in three amino acids during mRNA translation, that is, the 249th amino acid changes from serine (S) to leucine (L), the 290th amino acid from arginine (R) to serine (S), and the 347th amino acid from valine (V) to glycine (G). To further validate the mutations, resequencing analysis was also conducted in five additional melon lines (Supplementary Figure 1). When comparing the DNA sequence of these five lines, ZT091 and M2-10 exhibited the same sequence mutant with P5, while Bobo and ms5 had the same sequence as the reference genome sequence of parental line P10 (Supplementary Figure 1).

## Discussion

Different types of vegetable seeds are accompanied by dormancy or germination disorders. For example, melon seeds can remain dormant for more than 6 months (Lopez-Molina et al., 2001; Piskurewicz et al., 2016), Cucurbita moschata (Duch. ex Lam.) Duch. ex Poiret seeds have a germination rate of only ~40% within the same year, while other cucurbits, such as *Benincasa hispida* (Thunb.) Cogn., *Momordica charantia* L., *Citrullus lanatus* (Thunb.) Matsum. et Nakai, and *Cucumis melo* L. exhibit dormancy characteristics in some varieties. Seed germination is determined by genetic factors and environmental conditions (Agacka-Mołdoch et al., 2021). Seed survival s can be determined by changes in temperature during the seed germination process, which could affect plant survival rate and final yield production. When the seeds are germinated under high or low temperatures, seed dormancy increases, and the germination rate decreases (Bodrone et al., 2017). To delineate the genetic basis of germination in vegetables, several independent studies have been conducted using different conditions for seed germination, and several underlying QTLs have been identified (Jiang et al., 2017; Rehman Arif and Börner, 2020). Compared to other crops, not much research has been conducted on melon seed germination. Evaluating the main factors influencing *Cucumis melo* L. seed germination and determining the optimal combination of germination temperature and water immersion time can provide an important resource for the optimal seed propagation of *Cucumis melo* L.. Here, we investigated the dynamic trend of seed germination in *Cucumis melo* L. under different temperature and water immersion time combinations. Our results indicated that a longer immersion time led to a higher germination rate under the same temperature treatment. In addition, a higher treatment temperature significantly increased the germination rate under the same immersion time. However, no further effects were observed after reaching a certain temperature plateau. For the parental line P5 that exhibited seed germination disorders, the responses to immersion time and treatment temperature were not significant, with germination starting at 72 h with the above-mentioned treatments. This indicates a low correlation between moisture and temperature conditions, probably due to other germination disorders, specifically for this genotype.

Seed germination and dormancy are two complex and closely related physiological processes that are mainly regulated by qualitative genes (Xiang et al., 2014; Sato et al., 2016; Xu et al., 2020). The location of genes controlling seed germination traits has been extensively studied in other crops, with significant discoveries made in rice (Liu et al., 2016), wheat (Zheljazkov et al., 2021), and maize (Zhang et al., 2022a). In terms of *Cucumis melo* L., a QTL analysis for the seed size trait has already been conducted (Zhang et al., 2022b). Using genome-wide association analysis in *Solanum lycopersicum*, the *SIMAPK11* was identified to regulate seed dormancy, indicating that the ABA pathway was involved in seed dormancy and germination. In *Brassica*, 12 QTL loci were associated with seed germination, and among them, six contained genes homologous to previously identified seed dormancy genes in *Arabidopsis* (Wang et al., 2016). More recently, QTLs controlling low-temperature germination (LTG) ability were identified in cucumber, with a major effect QTL *qLTG1.2* discovered and narrowed to a 348 Kb region. In rice, more than 30 LTG QTLs have been identified, with unknown protein candidate genes for two of the QTLs (Yagcioglu et al., 2019). The results indicated that this gene is likely involved in germination (Fujino et al., 2008).

In our study, we used a SLAF-generated genetic map to locate genes of *Cucumis melo* L. underlying seed germination traits. The seeds were harvested in the same year, and genetic analysis indicated that seed germination is a typical quantitative trait, similar to other species, with its related traits being highly complex and under polygenic control. Based on QTL analysis in 2 consecutive years, a total of eight QTLs were detected in six chromosomes. QTL *2020-qsg4.1* was located in the genome position 2,073,104-2,609,033 within a 535.93 Kb interval, containing 53 functional genes. The *2020-qsg5.2* candidate region spans 47.18 Kb with seven candidate genes including E3 ubiquitin-protein ligase PRT1 genes related to seed development in Cucurbits (Guo et al., 2020). In the 2021 germination experiment, the QTL *2021-qsg2.1* spans 197.96 Kb and contains 26 candidate genes. Although two QTLs on chromosome 6 were detected in both years of experiments, they were not in the same region (Table 4). The QTL *2020-qsg6.1* region is located in the genome position between 4,541,536 and 7,591,934 bp, spanning 3.05 Mb, which is a relatively large interval, while *2021-qsg6.1* is located between727,434 and 874,956 bp, a 147.52 Kb interval. Potential candidate genes located in these regions were the jasmonic acid-amido synthetase JAR1 and ethylene-responsive transcription factor-related genes related to seed dormancy (Nguyen et al., 2018). The chromosome 8 QTL *2021-qsg8.1* is located between 1,742,525 and1,779,289 bp, a 36.26 Kb region that contains six functional genes. Among them, a potential candidate is a NAC domain-containing protein, which is well-known in plant growth, particularly in seed germination and dormancy (Mathew et al., 2016; Kong et al., 2020; Zhang et al., 2022c). Other potential candidate genes, such as the receptor-like protein kinase HSL1, were also located in the region. This gene was recently characterized and related to seed dormancy in wheat (Torada et al., 2016) and Arabidopsis (Chen et al., 2020; Wang and Gou, 2020). As these QTLs were only detected in one year, we cannot conclude firmly that they regulate the germination process in melon seeds. A more complex design including more markers or other populations to identify and confirm these QTLs should be conducted.

Among the QTLs detected in both years, only one stable consistent QTL *qsg5.1* was detected. The other QTLs detected on chromosomes 2, 4, 6, and 8 indicated that environmental factors influence the seed germination in melons. In 2020, seeds from F_2:3_ families were planted in Heilongjiang Province (latitude 125.03°, longitude 46.58°, and the average temperature was 25.3°C in the planting season). In contrast, in 2021, seeds were collected from F_2:3_ families in Hainan Province (latitude 109.50°, longitude 18.09°, and average temperate was 28.5°C). Thus, the different geographic locations and environmental conditions could influence the seed germination rate. With the high-density genetic map, the *2020-qsg5.1* QTL was located in a 33.83 Kb region containing only four genes, whereas the *2021-qsg5.1* QTL spanned 100.77 Kb, containing 15 candidate genes. This supports that the high-resolution genetic map could help toward a more precise QTL detection (Lv et al., 2022).

Although the candidate region was narrow, to further confirm that the *qsg5.1* was the candidate region for seed germination in the present study, seeds from 421 additional fruits of F_2_ individuals were used to narrow it further down and confirm the results. Based on the resequencing data from the parental lines P5 and P10 and a gene structure analysis, eight single nuclear polymorphic (SNP) loci and eight CAPS (markers were designed and investigated. The fine mapped candidate regions were between SNP53 and SNP54 within 5,374,017–5,424,974 bp. This region contains four genes, and among these, two have unknown functions (Table 5). Two genes did not show significant differences in gene expression. The transmembrane protein-related gene (*MELO3C031218.2*) was differentially expressed in parental line P10 but not in P5 in all three germination stages. Thus, this gene may control the parental line P10 germination stages. Another explanation for this interesting observation was that P5 did not germinate until 48 h after treatment. Thus, from 24 to 48, the gene might not be expressed. Extending the expression analyses to further time points could help understand if the transmembrane protein-related gene is associated with the control of seed germination in melons. *MELO3C031219.2* is differentially expressed in all germination stages; its functional annotation is a phosphate transporter (PHO) protein gene (Song, 2018). To detect the *MELO3C031219.2* expression pattern in other melon germination stages, an analysis of resequencing data by Wang et al. (2021) and Dai et al. (2022) was carried out. The results were consistent in P10 and the additional five melon lines evaluated. However, they were different in P5; therefore, these three mutations could be involved in delaying melon germination and should be further studied.

The results indicated three mutations in P5, M2-10, and ZT091, but absent in P10, Bobo, ms5, and 1,244 (the sequence of these genotypes was the same as the reference genome). These three mutations could attribute to the difficulty or delay in melon germination and should be further investigated. Interestingly, the genotypes P5, M2-10, and ZT091 had a relatively short growth period, with about 28 days to bear fruits after transplantation, and they belong to muskmelon. On the other hand, the genotypes Bobo and ms5 have a long growth period with about 45 days to bear fruits after transplantation, while genotype 1,244 was a semi-wild line. Therefore, deeper evolutionary relationships may be related to the single-nucleotide mutation discovered.

We further analyzed whether there are homologous genes in the candidate gene regions that can affect seed germination, through evolutionary-mediated variation. To identify the relationship between the candidate genes, a cluster analysis was conducted. Among these, the candidate gene *MELO3C031219.2* and one unknown gene *MELO3C031220.2* were nearly evolutionary identical, but others were not (Supplementary Figure 2). It is possible that these genes independently regulate seed germination traits. However, a specific functional analysis must be undertaken to validate this hypothesis.

In Arabidopsis, the PHO gene family contains 11 members, denoted as PHO:H1-H10 (Song, 2018). Huang et al. (2016) observed a reduced expression of the PHO1 gene after treating Arabidopsis seeds with ABA; the PHO1 protein was found to interact with the ABA insensitive 5 (ABI5) transcription factor involved in the inhibition of seed germination by ABA. Different components of the ABA signaling pathway have been shown to affect seed germination directly (Wang et al., 2016). The Arabidopsis PHO1:H4 protein also influences hypocotyl elongation (Cao et al., 2021). The PHO:H10 gene expression is induced by ABA and the JA synthetic precursor 12-oxo-phytodienoic acid, suggesting that the function of the PHO:H10 gene is related to stress and hormone response with important physiological functions. In rice, a member of the phosphate transporters (PTs) Pht1 family (OSPT8) was found to play a role in plant development through the regulation of Pi signal transduction (Dong et al., 2019).

To conclude, a total of eight QTLs of seed germination in melons were detected using a SLAF genetic map, and among them, QTL *qsg5.1* was the most consistent. A major candidate gene for *qsg5.1* based on differential expression was also identified. The results provide a theoretical basis for melon breeding toward optimizing melon seed germination.

## Conclusion

Using 358 F_2:3_ families from two diverse parents, based on the constructed high-density genetic map of C. melo using SLAF sequences, a total of eight QTLs for seed germination traits in 2 years were identified. A consistent stable major QTL *qsg5.1* was identified. An additional 421 F_2_ plants were used to fine map QTL *qsg5.1* with CAPS markers. The results indicated that four candidate genes are located within the 55.96 Kb QTL region. Based on gene expression and SNP analysis, the *MELO3C031219.2* (phosphorus transporter PHO-5 family proteins) gene was the strongest candidate that was linked to seed germination regulation. The results provide the basis for mapping-based gene cloning and MAS breeding for germination-related traits in melons.

## Data availability statement

The original contributions presented in the study are publicly available. This data can be found here: https://www.ncbi.nlm.nih.gov/bioproject/PRJNA812343.

## Author contributions

YS designed this experiment. LW and JL performed the research and wrote the manuscript. FY and DD conducted data analysis. XL collected phenotypic data in field trials. All authors reviewed and approved the manuscript before submission.

## Funding

This study was supported by the National Natural Science Foundation of the People's Republic of China (No. 31772330), the Natural Science Foundation of the Heilongjiang Province (No. LH2021C068), the Heilongjiang Bayi Agricultural University Support Program for San Zong (TDJH202004), and Daqing Guiding Science and Technology Plan Project (zd-2020-66).

## Conflict of interest

The authors declare that the research was conducted in the absence of any commercial or financial relationships that could be construed as a potential conflict of interest.

## Publisher's note

All claims expressed in this article are solely those of the authors and do not necessarily represent those of their affiliated organizations, or those of the publisher, the editors and the reviewers. Any product that may be evaluated in this article, or claim that may be made by its manufacturer, is not guaranteed or endorsed by the publisher.

## Supplementary material

Supplementary Figure 1 DNA sequences analysis of P5, P10, and other five melon germplasms of candidate gene *MELO3C031219.2*.

Supplementary Figure 2 Cluster analysis of identified candidate and other genes at the *qsg5.1* locus.

Supplementary Table 1 Primer information of candidate region in *qsg5.1* on chromosome 5.
