# Supplementary figures and images for: A preliminary mapping of QTL qsg5.1 controlling seed germination in melon (Cucumis melo L.)
Source: Front Plant Sci. 2022 Aug 15;13:925081. doi: 10.3389/fpls.2022.925081 (PMC9421157; doi:10.3389/fpls.2022.925081)

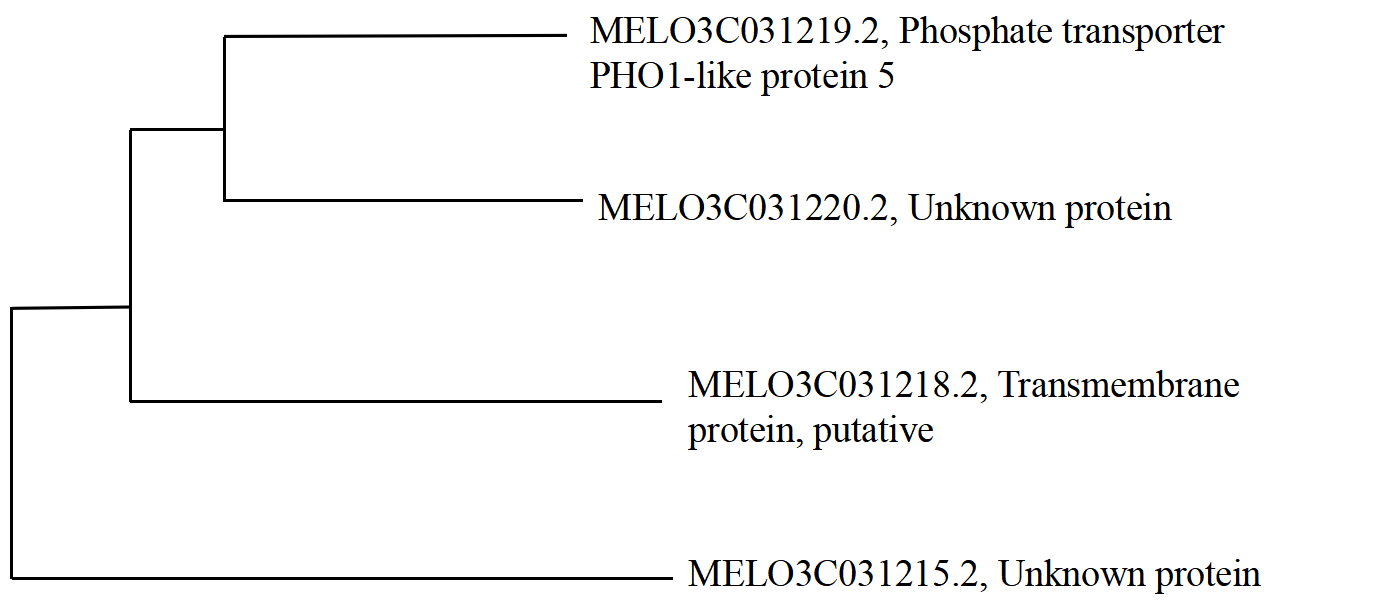

Supplement: Supplementary Figure 1 — DNA sequences analysis of P5, P10, and other five melon germplasms of candidate gene MELO3C031219.2. [file Image_1.TIF]

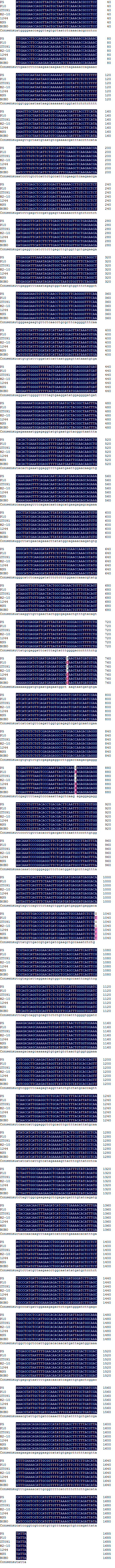

Supplement: Supplementary Figure 2 — Cluster analysis of identified candidate and other genes at the qsg5.1 locus. [file Image_2.TIF]
